# Classification of patients with bipolar disorder using k-means clustering

**DOI:** 10.1371/journal.pone.0210314

**Published:** 2019-01-23

**Authors:** Lorena de la Fuente-Tomas, Belen Arranz, Gemma Safont, Pilar Sierra, Monica Sanchez-Autet, Ana Garcia-Blanco, Maria P. Garcia-Portilla

**Affiliations:** 1 Centro de Investigación Biomédica en Red de Salud Mental (CIBERSAM), Instituto de Salud Carlos III, Fondos FEDER, Madrid, Spain; 2 Department of Psychiatry, University of Oviedo, Oviedo, Spain; 3 Parc Sanitari Sant Joan de Deu and University of Barcelona, Barcelona, Spain; 4 University Hospital Mutua Terrassa and University of Barcelona, Barcelona, Spain; 5 La Fe University and Polytechnic Hospital and University of Valencia, Valencia, Spain; Department of Psychiatry and Neuropsychology, Maastricht University Medical Center, NETHERLANDS

## Abstract

**Introduction:**

Bipolar disorder (BD) is a heterogeneous disorder needing personalized and shared decisions. We aimed to empirically develop a cluster-based classification that allocates patients according to their severity for helping clinicians in these processes.

**Methods:**

Naturalistic, cross-sectional, multicenter study. We included 224 subjects with BD (DSM-IV-TR) under outpatient treatment from 4 sites in Spain. We obtained information on socio-demography, clinical course, psychopathology, cognition, functioning, vital signs, anthropometry and lab analysis. Statistical analysis: k-means clustering, comparisons of between-group variables, and expert criteria.

**Results and discussion:**

We obtained 12 profilers from 5 life domains that classified patients in five clusters. The profilers were: Number of hospitalizations and of suicide attempts, comorbid personality disorder, body mass index, metabolic syndrome, the number of comorbid physical illnesses, cognitive functioning, being permanently disabled due to BD, global and leisure time functioning, and patients’ perception of their functioning and mental health. We obtained preliminary evidence on the construct validity of the classification: (1) all the profilers behaved correctly, significantly increasing in severity as the severity of the clusters increased, and (2) more severe clusters needed more complex pharmacological treatment.

**Conclusions:**

We propose a new, easy-to-use, cluster-based severity classification for BD that may help clinicians in the processes of personalized medicine and shared decision-making.

## Introduction

Bipolar disorder (BD) is an episodic, often chronic, affective disorder frequently associated with functional impairment [1], excess morbidity [2], and premature mortality [3]. BD has been defined as a pleomorphic condition with a diversity of patterns and trajectories throughout its natural course [4], thus needing personalized decisions shared with patients [5] regarding treatment. Despite the aforementioned, current classifications are based mainly on psychopathological and functional information, neglecting the impact of this disorder on other dimensions of patients' lives and their subjective preferences, which limits their usefulness. In the last decade, staging models have been proposed [6–9] as an alternative that is better adapted to the processes of personalized medicine and shared decision-making. These models provide an alternative framework where mental disorders are placed on a probabilistic continuum from an at-risk or latent stage to late or end-stage disease [10].Thus, they would help guide clinicians in the selection of intervention approaches based on the possibility of disorder progression, as opposed to focusing on diagnostic category alone [11].

Several scientific literature on BD notes that is a progressive condition that would benefit from a staging model [12–13], particularly based on the concept of allostasis and allostatic load [12, 14], although there is no conclusive evidence. In addition, several studies have demonstrated the neurotoxicity [12, 15] of the disorder, leading to greater frequency and severity of episodes, greater sensitivity to adverse life events, and less time in euthymia. The raising interest in this subject is demonstrated by the increasing number of papers, among them some recent systematic reviews of great interest [16–18].

The first staging model of BD was proposed by Berk et al. [4] in 2006. They proposed 5 clinical stages (from 0—increased risk to 4—persistent disorder) based on psychopathology. In 2009, Kapczinski et al. [19–20] suggested a similar model, with the addition of functioning, cognitive performance, and blood biomarkers. Subsequently, Cosci and Fava [21] proposed an integrative model that emphasized the lack of evidence supporting a stage 0 (at-risk). Duffy et al. [22] later suggested a staging model that took into account the natural history of BD and the heterogeneity of subtypes.

While the models proposed are interesting, they only partially address the clinical reality of BD since they do not include other life domains that are relevant and may be affected in the course of the disorder, such as physical health status [23–24] and health-related quality of life [25–26], and all of them were theoretically developed. However, recently, an increasing number of authors are testing the behavior of different parameters in these theoretically proposed models. Thus, concerning peripheral blood inflammatory biomarkers, a decrease in IL-6, BDNF, and SIL-2R along with an increase in TNF-α, sTNFR80, MMP9 and sICAM-1 have been described in late stages by different authors [27–29] while an increase of IL-10 has been described in early stages [30]. Although promising, these results are preliminary and in some cases contradictory as demonstrated by the fact that Grande et al [31] found increased levels of IL-6 in late stage. Also, neuroanatomical anomalies and a reduction of the hippocampal volume have been described in late stages [32–33]. Cognition and functioning were also employed to differentiate among the different stages proposed. Contrary to biomarkers, the results found showed great concordance and indicated a progressive decline in cognition and functioning in late stages [31, 34–36], thus supporting the construct of staging in BD.

Despite the high interest on the area, Alda and Kapczinski [37] point out that most of the proposed models have not been tested, and the ISBD Task Force Report specifies that more efforts are needed to determine the details of the stages and how many are optimal [13]. In the meanwhile, we aim to develop a cluster-based classification, which reflects the different severity degrees of the disorder, using, in addition to the psychopathology of BD, cognition, physical health status, real-world functioning, and subjective health-related quality of life as relevant information for assigning each patient to a specific cluster.

## Methods

### Study design

Naturalistic, cross-sectional, multicenter study of patients with BD in ambulatory treatment. A total of 200 patients will allow us to classify subjects in different clusters based on a minimum of 8 variables.

The study was conducted at 4 sites in Spain [Oviedo (1 center), Barcelona (2 centers), and Valencia (1 center)] between January 2012 and December 2014. The Clinical Research Ethics Committee of Hospital Universitario Central de Asturias in Oviedo approved the study protocol (Ref. 36/2012). Written informed consent was obtained from all subjects prior to enrolment.

### Participants

Subjects were patients with a diagnosis of BD. Diagnoses were confirmed using the Structured Clinical Interview for DSM-IV Axis I Disorders (SCID-I) [38]. Inclusion criteria were (1) outpatients with a confirmed DSM-IV-TRN [39] diagnosis of BD in treatment at any of the 4 participating centers; (2) age ≥18 years; and (3) written informed consent to participate in the study.

Exclusion criteria were designed to be minimal in order to obtain a heterogeneous and representative sample of all phases of the disorder. Thus, we excluded only individuals who refused to participate in the study.

All patients who consecutively attended their regular appointments at any of the 4 study sites were offered to participate in the study if they met all the inclusion criteria and none of the exclusion criteria. A total of 224 patients fulfilled this requirement and agreed to participate giving their written informed consent. No patient was legally incapacitated, and their ability to give informed consent was determined by their psychiatrists.

### Assessments

We collected demographic and clinical data for all subjects and made comprehensive assessments at baseline. In addition to the classical variables, sociodemographic data included some pragmatic variables, such as number of school grades repeated, number of children and their custody, driver’s license, legal problems, officially recognized disability, etc. The clinical course and characteristics of BD, psychiatric and physical comorbidities, and treatments were also collected (Table 1).

**Table 1:** Patient sociodemographic and clinical characteristics.

**Sociodemographic characteristics**	
Mean age [Mean (SD)]	47.1 (12.5)
Gender, females [n (%)]	146 (65.2)
Marital status [n (%)]	
Never married	73 (32.6)
Married or living with partner / widowed	105 (46.9)
Separated / divorced	46 (20.5)
Educational level [n (%)]	
Primary school	61 (27.2)
Secondary school	110 (49.1)
University	53 (23.7)
Years of education [Mean (SD)]	13.0 (4.7)
Years of education repeated [Mean (SD)]	0.6 (0.8)
Children, yes [n (%)]	132 (58.9)
Number of children [Mean (SD)]	1.7 (0.8)
Custody of children [n (%)]	20 (19.2)
Patient	
Work status [n (%)]	49 (21.9)
Working (full/part-time)	36 (16.1)
Unemployed	99 (44.2)
Disabled due to bipolar disorder	40 (17.9)
Other*	77 (34.4)
Recognized disability, yes [n (%)]	159 (71)
Driver’s license, yes [n (%)]	30 (13.4)
Legal problems, yes [n (%)]	31 (13.9)
Risky sexual behavior, yes [n (%)]	
**Bipolar disorder characteristics**	**Mean (SD)**
Type of bipolar disorder [n (%)]	
Type 1	159 (71.3)
Type 2	61 (27.4)
Other	3 (1.3)
Dominant polarity [n (%)]	
Depressive	89 (40.6)
Manic/hypomanic	94 (42.9)
Mixed	36 (16.4)
Current episode [n (%)]	
Depressive	112 (50.0)
Manic	91 (40.6)
Hypomanic	19 (8.5)
Mixed	2 (0.9)
Length of illness, years [Mean (SD)]	19.4 (12.0)
Length since BD diagnostic, years [Mean (SD)]	11.6 (10.0)
Age at first psychiatric contact [Mean (SD)]	28.8 (11.3)
Age at illness onset [Mean (SD)]	27.8 (9.6)
Age at illness diagnosis [Mean (SD)]	35.5 (11.6)
Number of hospitalizations [Mean (SD)]	2.2 (2.6)
Age at first hospitalization [Mean (SD)]	33.3 (11.3)
Suicide attempts	
Yes [n (%)]	76 (33.9)
Number [Mean (SD)]	0.74 (1.6)
Number of depressive episodes [Mean (SD)]	3.6 (5.3)
Number of dysthymic episodes [Mean (SD)]	0.7 (1.6)
Number of manic episodes [Mean (SD)]	2.3 (3.9)
Number of hypomanic episodes [Mean (SD)]	1.8 (2.5)
Number of mixed episodes [Mean (SD)]	1.1 (3.1)
Comorbid personality disorder [n (%)]	41 (18.3)
Comorbid substance use disorder [n (%)]	26 (11.6)
Comorbidity with other mental disorder [n (%)]	53 (23.7)
Use of substances [n (%)]	
Tobacco (current)	100 (44.6)
Cigarettes per day [Mean (SD)]	16.6 (9.5)
Alcohol (current)	51 (22.8)
Cannabis (lifetime)	36 (16.1)
Cocaine (lifetime)	17 (7.6)
Family psychiatric history [n (%)]	
Affective disorder	111 (49.6)
Other mental disorders	28 (12.5)
No	85 (37.9)
Age of father at birth [Mean (SD)]	32.1 (6.9)
Age of mother at birth [Mean (SD)]	29.2 (6.6)
**Psychometric instruments**	**Mean (SD)**
Clinical Global Impression, Severity (CGI-S)	3.4 (3.8)
@Hamilton Depression Rating Scale (HDRS)	7.3 (6.0)
#Hamilton Anxiety Rating Scale (HARS)	9.6 (7.5)
#Young Mania Rating Scale (YMRS)	2.6 (3.6)
Oviedo Sleep Questionnaire (OSQ)	
Satisfaction with sleep	4.5 (1.7)
Changes in Sexual Functioning Questionnaire (CSFQ)	33.2 (11.6)
Scale for Cognitive Impairment in Psychiatry (SCIP)	2.9 (1.6)
Functioning Assessment Short Test (FAST)	
Global score	26.3 (16.1)
Autonomy	3.5 (3.4)
Occupational functioning	8.1 (5.7)
Cognitive functioning	6.0 (4.1)
Financial issues	1.3 (1.8)
Interpersonal relationships	5.2 (4.2)
Leisure time	2.5 (2.0)
Global Assessment of Functioning (GAF)	68.0 (15.3)
The MOS 36-item Short-Form Health-Survey (SF-36) z-scores	
Physical functioning	-0.4 (1.1)
Physical role	-0.6 (1.3)
Bodily pain	-0.3 (1.1)
General health	-0.6 (1.2)
Vitality	-0.9 (1.0)
Social functioning	-1.3 (1.7)
Emotional role	-0.9 (1.7)
Mental health	-0.6 (1.2)
Physical Summary Component	46.4 (10.8)
Mental Summary Component	39.6 (15.3)
**Laboratory results**	**Mean (SD)**
Hematology	
Erythrocytes	4.5 (0.4)
Hemoglobin	14.0 (1.4)
Leukocytes	7.4 (2.4)
Platelets	245.4 (69.2)
Lipid profile	
Cholesterol	203.8 (42.8)
LDL cholesterol	119.5 (38.3)
HDL cholesterol	54.5 (14.6)
Triglycerides	150.3 (94.2)
Glucose	93.6 (23.3)
Hepatic function	
GPT	22.1 (17.6)
GOT	22.7 (17.0)
GGT	35.2 (62.1)
Bilirubin	0.6 (0.3)
Renal function	
Creatinine	0.8 (0.2)
BUN	32.8 (9.9)
Hormones	
PRL	24.5 (28.5)
TSH	12.7 (2.7)
Inflammatory and oxidative biomarkers	
CRP	1.6 (3.2)
Homocysteine	13.4 (5.9)

* Other includes: disabled due to other illness, student, homeworker and retired.

@Bipolar cut-off scores: HDRS: 0–7 = no depression, ≥8 = depression.

#Spanish cut-off scores:—HARS: 0–5 = no anxiety, 6–14 = mild anxiety, ≥15 = moderate/severe anxiety,—YMRS: ≤6 = euthymia, 7–20 = mixed episode, >20 = manic episode

Abbreviations: SD = standard deviation; GPT = Glutamyl pyruvic transaminase; GOT = Glutamyl oxaloacetic transaminase; GGT = Gamma-glutamyl transferase; BUN = Blood urea nitrogen; PRL = Prolactin; TSH = Thyroid-stimulating hormone; CRP = C-reactive protein.

Psychometric assessments were done using the Spanish versions of all the following instruments: (1) Psychopathology: Hamilton Depression Rating Scale (HDRS) [40], Young Mania Rating Scale (YMRS) [41], Hamilton Anxiety Scale (HARS) [42], Oviedo Sleep Questionnaire (OSQ) [43], Changes in Sexual Functioning Questionnaire Short-Form (CSFQ-14) [44], (2) Cognition: Scale for Cognitive Impairment in Psychiatry (SCIP) [45], (3) Real-world functioning: Functioning Assessment Short Test (FAST) [46] and the Global Assessment of Functioning (GAF) [47], (4) Health-related quality of life: The MOS 36-item Short-Form Health-Survey (SF-36) [48], and (5) Global severity: Clinical Global Impression, Severity (CGI-S) [49].

Clinical examinations included: anthropometry (height, weight, and waist circumference), vital signs (heart rate and blood pressure), and laboratory tests including hematology and biochemistry, hormones, and biomarkers of inflammation and oxidative stress (for complete information, see Table 1). Venous blood samples (10 mL) were collected between 8:00 and 10:00 am after fasting overnight. Body Mass Index (BMI) was calculated as body weight (kg) divided by height squared (m^2^). The presence of metabolic syndrome was determined according to the NHANES 1999–2000 criteria [50].

### Statistical analyses

The statistical analyses were done using SPSS 17.0. A dimensional reduction was done using k-means clustering. This technique aims to partition n observations into k clusters in which each observation belongs to the cluster with the nearest mean. Comparisons of between-group variables were then performed by Chi-square and univariate ANOVA followed by Tukey’s honestly significant difference post-hoc testing. Those variables in which statistically significant differences between groups were found were selected to be part of the model along with other variables added by expert criteria. We used all these variables, hereafter called profilers, to calculate a global severity formula. We then calculated the 5th, 25th, 50th, 75th, and 95th percentiles of the score in this formula to propose our severity clusters.

To determine the construct validity of the proposed clusters, we employed the scores on the 12 profilers and the patient pharmacological treatment patterns (number of drugs, numbers of patients on stabilizers, antipsychotics, antidepressants, and benzodiazepines) as validator variables. The hypothesis is that, in the more severe clusters, patients will obtain more severe scores on all the profilers and will require highly complex pharmacological treatments. We used the ANOVA (Tukey’s honestly post hoc) and Chi-square tests for that purpose.

## Results

In all, 224 patients were enrolled: 101 (45.1%) from Mental Health Centers I and II in Oviedo, 23 (10.3%) from Parc Sanitari Sant Joan de Deu in Barcelona, 50 (22.3%) from Hospital Universitari Mútua Terrasa in Barcelona, and 50 (22.3%) from Hospital Universitario La Fe in Valencia.

### Demographic and clinical characteristics

The demographic and clinical characteristics of the sample are shown in Table 1. Mean age was 47.1 years, 65.2% were female, 46.9% were married, and 21.9% were working. Seventy-two percent of the patients had from type 1 BD and the mean time interval between illness onset and diagnoses was 6.5 (SD = 11.3) years. Comorbidity with personality and substance use disorders was found in 18.3% and 11.6% of patients, respectively. Regarding use of substances, tobacco was the most commonly used (44.6%), followed by alcohol (22.8%). Family history of affective disorders was positive in 49.6% of the sample (for further details, see Table 1).

The mean CGI-S score was 3.4 (SD = 3.8) (see Table 1). Of the 224 subjects at the time of assessment, 39.7% had a score consistent with depression according to the HDRS (≥7), 12.1% with a mixed episode (YMRS ≥ 7–20), and only one subject with a manic episode according to the YMRS (>20). Concerning cognition, 23.2% had mild, 25.9% moderate, and 15.5% severe impairment. On average, patients were receiving 2.9 (SD = 1.1) drugs for treatment of their BD at enrolment in the study. Of the 224 patients included, 192 were receiving mood stabilizers [155 (69.2%) were taking one and 37 (16.5%) two], 149 patients (66.5%) antipsychotics [125 (55.8%) one and 24 (10.7%) two], 88 patients (39.3%) antidepressants, and 122 (54.5%) benzodiazepines.

Concerning physical health, the mean BMI was 28.5 (SD = 5.3) kg/m^2^, and 36.2% patients were obese (BMI ≥ 30). In addition, 58 (26.7%) patients had metabolic syndrome. Table 1 shows the laboratory results.

### Development of the global severity formula and clusters

Using k-means clustering, comparisons of between-group variables, and expert criteria, 12 profilers from 5 life domains were selected to form part of the global severity formula. The 12 profilers (all with the same weight) are: (1) Clinical characteristics of the BD: 3 profilers: Number of hospitalizations (HospN), Number of suicide attempts (SuicAttN), and Comorbid personality disorder (ComPD); (2) Physical health: 3 profilers: Body Mass Index (BMI), Metabolic Syndrome (MetS), and Number of comorbid physical illnesses (IllnessN); (3) Cognition: 1 profiler: Screen for Cognitive Impairment in Psychiatry score (SCIP-Tr4); (4) Real-world functioning: 3 profilers: Permanently disabled due to BD (PDxBD), Functioning Assessment Short Test total score (FAST-T), and Functioning Assessment Short Test leisure time subscale score (FAST-leisure); and (5) Health-related quality of life: 2 profilers: SF-36 physical functioning scale score (SF-PF), and SF-36 mental health scale score (SF-MH). Profilers in the formula can take values from 0 to 1 (Table 2).

**Table 2:** Variables in the global severity formula and their given values.

Variable name	Given value
Number of hospitalizations	No hospitalization = 0; 1 = 0.2; 2 = 0.4; 3 = 0.6; 4 = 0.8; ≥5 = 1
Number of suicide attempts	No attempts = 0; 1–2 attempts = 0.33; 3–4 = 0.66; ≥5 = 1
Comorbid personality disorder	Yes = 1; No = 0
Body mass index	BMI < 25 = 0; 25 ≤ BMI < 30 = 0.25; 30 ≤ BMI < 35 = 0.50; 35 ≤ BMI < 40 = 0.75; BMI ≥ 40 = 1
Metabolic syndrome	Yes = 1; No = 0
Number of comorbid physical illnesses	No illness = 0; 1 illness = 0.25; 2 = 0.5; 3 = 0.75; ≥4 = 1
SCIP total score	No cognitive impairment = 0; mild = 0.33; moderate = 0.66; severe = 1
Permanently disabled due to bipolar disorder	Yes = 1; No = 0
FAST total score	Value of FAST total score ranges from 0 to 66 (discounting the FAST leisure time subscale score). Value for the formula is total score divided by 66.
FAST leisure time subscale score	Value for the formula is the subscale score divided by 6.
SF-36 physical functioning scale score	< -2 sd = 1; -2 to +2 sd = 0.5; > +2 sd = 0
SF-36 mental health scale score	< -2 sd = 1; -2 to +2 sd = 0.5; > +2 sd = 0

Abbreviations: BMI = Body mass index; FAST = Functioning Assessment Short Test; SCIP = Scale for Cognitive Impairment in Psychiatry; sd = standard deviation; SF-36 = The MOS 36-item Short-Form Health-Survey.

The sum of all profilers is multiplied by 10/12 in order to obtain a global severity score that ranks severity from 0 (low) to 10 (high). The formula is:

$$Severity\;=\;\frac{10}{12}\times \left(PDxBD+MetS+ComPD+SCIP-Tr4+IllnessN+SF-PF+SF-MH+FAST-T+FAST-leisure+BMI+HospN+SuicAttN\right)$$

The mean global severity score was 3.6 (SD = 1.4), with a minimum value of 0.8 and maximum of 8.0. As can be seen, data follows a normal univariate distribution with Skewness and Kurtosis values of 0.440 and -0.158, respectively.

In Table 3, we show the global severity score ranges corresponding to the 5th, 25th, 50th, 75th, and 95th percentiles. Thus, a global severity score ranging from 0 to 1.70 falls between the 0 and 5th percentiles, corresponding to the first cluster, called “Not widespread, effect limited to symptoms of BD.” Similarly, global severity scores greater than 6.10 belong to cluster 5, percentiles equal to or greater than 95, called “Globally widespread, all five life domains are affected.” Following this, 13 patients (5.8%) were classified as cluster 1, 43 (19.2%) as cluster 2, 108 (48.2) as cluster 3, 47 (21.0) as cluster 4, and 13 (5.8%) as cluster 5.

**Table 3:** Percentiles, severity score range values, and proposed clusters.

Percentile	Severity score range	Proposed cluster
5	0.00–1.70	1. Not widespread, effect limited to symptoms of bipolar disorder
25	1.71–2.50	2. Mildly widespread
50	2.51–4.50	3. Moderately widespread
75	4.51–6.10	4. Extensively widespread
95	≥ 6.11	5. Globally widespread, all five life domains affected

### Construct validity of the proposed clusters

As can be seen in Table 4, all 12 profilers behave correctly, significantly increasing in severity as the severity of the clusters increases, thus providing proof of construct validity.

**Table 4:** Values and distribution of variables in the classification across the five proposed clusters.

Life area variables	Cluster 1 (n = 13)	Cluster 2 (n = 43)	Cluster 3 (n = 108)	Cluster 4 (n = 47)	Cluster 5 (n = 13)	Statistical test, *p*
**Bipolar disorder**						
**Number of hospitalizations [Mean (SD)]**	0.8 (0.6)	1.9 (2.6)	2.0 (2.5)	2.9 (2.4)	4.2 (3.1)	4.397,^1^ 0.002
**Number of suicide attempts [Mean (SD)]**	0.4 (0.8)	0.7 (1.6)	0.6 (1.1)	0.9 (1.3)	2.1 (4.2)	3.008,^1^ 0.019
**Comorbid personality disorder, yes [n (%)]**	0 (0.0)	1 (2.3)	17 (15.7)	14 (29.8)	9 (69.2)	37.421,^2^ <0.0005
**Physical health**						
**Body Mass Index [Mean (SD)]**	24.2 (3.4)	24.9 (3.1)	27.7 (4.5)	32.3 (3.7)	36.2 (7.0)	30.902,^1^ <0.0005
**Metabolic Syndrome, yes [n (%)]**	1 (7.7)	5 (12.5)	28 (26.7)	19 (41.3)	5 (38.5)	12.445,^1^ 0.014
**Number of comorbid physical illnesses [Mean (SD)]**	0.5 (0.5)	0.5 (0.7)	1.1 (1.3)	1.7 (1.9)	2.2 (2.0)	7.851,^1^ <0.0005
**Cognition**						
**SCIP category, no cognitive impairment [n (%)]**	12 (92.3)	22 (51.2)	37 (35.6)	7 (14.9)	0 (0.0)	55.176,^2^ <0.0005
**Real-world functioning**						
**Permanently disabled due to bipolar disorder, yes [n (%)]**	0 (0.0)	2 (4.7)	46 (42.6)	39 (83.0)	12 (92.3)	78.537^2^, <0.0005
**FAST total score [Mean (SD)]**	5.9 (6.1)	13.5 (7.8)	26.8 (14.3)	36.4 (13.4)	47.8 (10.2)	37.200,^1^ <0.0005
**FAST leisure subscale score [Mean (SD)]**	0.5 (0.8)	1.1 (1.2)	2.6 (1.8)	3.5 (1.9)	4.5 (1.7)	20.712,^1^ <0.0005
Health-related quality of life*						
**SF-36 physical functioning scale score [Mean (SD)]**	0.4 (0.4)	-0.1 (0.7)	-0.3 (1.0)	-0.8 (1.5)	-1.2 (1.3)	7.216,^1^ <0.0005
**SF-36 mental health scale score [Mean (SD)]**	0.1 (0.7)	0.1 (1.0)	-0.7 (1.1)	-0.9 (1.3)	-0.6 (1.2)	7.257,^1^ <0.0005

Abbreviations: FAST = Functioning Assessment Short Test; SCIP = Scale for Cognitive Impairment in Psychiatry; SD = standard deviation; SF-36 = The MOS 36-item Short-Form Health-Survey.

*. Standardized scores.

1. ANOVA test;

2. Chi-square test.

A second strategy to test the construct validity of our cluster-based classification was to study the pharmacological treatment patterns across clusters. We found that, while clusters 1 and 2 were associated with monotherapy or use of two or three drug combinations, clusters 3, 4, and 5 were highly associated with combinations of four or more drugs (see Table 5). Furthermore, late clusters were associated with the use of two mood stabilizers, antipsychotics, antidepressants, and benzodiazepines more frequently than early clusters (Table 5).

**Table 5:** Distribution of prescription patterns among the five proposed clusters.

	Cluster 1 (n = 13)	Cluster 2 (n = 43)	Cluster 3 (n = 108)	Cluster 4 (n = 47)	Cluster 5 (n = 13)	Chi-square test, *p*
**Number of prescribed psychopharmaceuticals [n (%)]**						7.209, <0.0005
** Patients on One drug**	4 (30.8)	6 (14.0)	9 (8.3)	3 (6.4)	1 (7.7)	
** Two drugs**	3 (23.1)	19 (44.2)	25 (23.1)	8 (17.0)	0 (0.0)	
** Three drugs**	6 (46.2)	11 (25.6)	36 (33.3)	23 (48.9)	3 (23.1)	
** Four drugs**	0 (0.0)	7 (16.3)	28 (25)	8 (17.0)	5 (38.5)	
** Five or more drugs**	0 (0.0)	0 (0.0)	9 (8.3)	5 (10.6)	4 (30.8)	
**Type of prescribed psychopharmaceuticals [n (%)] Patients on Stabilizers**						2.375, 0.053
** One drug**	12 (92.7)	33 (82.6)	76 (70.4)	29 (61.7)	5 (38.5)	
** Two drugs**	0 (0.0)	4 (9.3)	17 (15.7)	9 (19.1)	7 (53.8)	
** Antipsychotics**	6 (46.2)	23 (53.5)	72 (66.7)	39 (85.1)	8 (61.5)	3.790, 0.005
** Antidepressants**	5 (38.5)	10 (23.3)	47 (43.5)	17 (36.2)	9 (69.2)	10.526, 0.032
** Benzodiazepines**	4 (30.8)	17 (39.5)	64 (59.3)	27 (57.4)	10 (76.9)	10.621, 0.031

## Discussion

Our study aimed to provide a cluster-based severity classification of patients with bipolar disorder using in addition to psychopathology and cognition other life domains such as physical health, functioning and quality of life. As previously hypothesized, our classification includes other relevant life areas that may be affected in patients with bipolar disorder, such as physical health and quality of life. Therefore, we propose a five-cluster classification that ranges from the least severity (only the psychopathological dimension is affected) to the most severe cluster in which other dimensions of life such as physical health, cognition, functioning and quality of life are also altered. Furthermore, the proposed classification successfully passes the preliminary test for construct validity, as all profilers significantly get worse scores from cluster 1 to 5 and patients needed more complex pharmacological treatments in the more severe clusters.

It is necessary to point out, that some of our life dimensions have also been proposed by other authors, with cognition [20–21, 34–35] and functioning [20–21, 32, 36–37] the most consistently proposed. However, we are unique in deconstructing life dimensions in profilers and, most importantly, in providing a way to measure them, as can be seen in Table 3. Thus, our three functioning profilers include a pragmatic variable, “being permanently disabled due to bipolar disorder,” and two psychometric scores, the total FAST and its leisure time subscale. It may be seen as a weakness that the model does not include the working subscale. However, from a psychometric point of view, the permanently disabled profiler gathers this type of information more accurately than the FAST working subscale. One unique profiler represents the cognitive dimension and classifies patients according to their SCIP score as having no cognitive impairment or mild, moderate, or severe cognitive impairment. Regarding clinical characteristics, number of previous episodes [36, 51–52], illness severity [35], and treatment response [53–55] have been proposed by different authors. Our formula includes three profilers in this dimension that better reflect illness severity: lifetime number of hospitalizations and suicide attempts, and presence of comorbid personality disorders. Finally, there are the two other dimensions included in our classification formula: physical health and quality of life. The physical health dimension involves three profilers: BMI, presence of metabolic syndrome, and number of comorbid physical illnesses. It should be noted that, although we included several blood biomarkers in the study, none of them were included in the model. This is especially surprising in the case of PCR, an inflammatory biomarker, since some authors have suggested a dysregulation of the immune response and a proinflammatory state in these patients [56]. We think this may be related to the classification formula’s inclusion of the metabolic syndrome, a pro-inflammatory state that could be redundant with inflammatory blood biomarkers, thus prevailing over them in the model. Lastly, the SF-36 scales of physical functioning and mental health are the two profilers of the quality of life dimension. The formula’s inclusion of the physical functioning scale is in keeping with the inclusion of the physical health dimension and reflects the great importance that patients themselves place on their physical condition.

We preliminarily tested the construct validity of our cluster-based severity classification by using the 12 profiler scores and the patient pharmacological treatment patterns. As hypothesized, we were able to demonstrate that patients belonging to the more severe clusters, reflecting a greater effect of the illness on their lives, received significantly worse scores in all the profilers than those allocated to the mildest clusters. The classification fits best in three life domains: physical health, functioning, and cognition. Regarding physical health, patients in more severe clusters had higher BMI, more physical comorbidities, and a greater proportion had metabolic syndrome than those in mildest clusters. Regarding functioning and cognition, from cluster 1 to cluster 5, we found a significant decline in total and leisure subscale FAST scores and in the percentage of patients who showed cognitive impairment. These results support the theoretical models proposed by Kapczinski et al. [20–21] and Grande et al. [32] and confirm the previous findings by Rosa et al. [37] which suggested functioning and cognition as markers that might help classify patients into different clinical stages of BD.

Further evidence of the construct validity of our cluster-based classification was the fact that the pattern of pharmacological treatment becomes more complex in the more severe clusters, with greater numbers and more types of drugs per patient compared to the mildest clusters. These results are in line with the theoretical hypothesis [57] and a recently published study [54].

To finalize, we would like to highlight that, since Passos and Kapczinsky [58] noticed that staging models currently available are not ready for clinical use, our cluster-based severity classification could be a first step that would help clinicians to classify, monitor and provide structured health care to their patients as a way to guarantee the quality of services [57]. Furthermore, as the profilers needed for doing so are very easy to obtain in daily clinical practice it could be implemented in the majority of the facilities dealing with patients with bipolar disorder.

Our results should be interpreted in light of one main limitation. We failed to include extremely severe patients according to our classification. Thus, the global severity score obtained, although normally distributed, was slightly left-skewed. The fact that one of the inclusion criteria was being an outpatient may have contributed to this failure. Furthermore, as subjects in the study had to have a diagnosis of bipolar disorder, prodromal phases were not included either. Also, further research should be conducted regarding two issues: first, to confirm the construct validity of the cluster-based classification using a completely different sample of subjects, and secondly, to determine if our assumption that all 12 profilers contribute with equal weight to global severity score is correct.

The main strength of our study is its empirical approach, that is, severity clusters were derived mathematically from clinical data rather than theoretically defined. Furthermore, our proposed classification includes relevant information from multiple life dimensions and precisely describes how to obtain and measure its 12 profilers. Moreover, these profilers are easy for clinicians to obtain in their daily clinical practice as they do not involved sophisticated techniques or additional costs. Another important point is the sample size and the exhaustive psychometric and clinical evaluations performed.

In conclusion, we propose a new, cluster-based severity classification that may improve current classifications in helping clinicians in the processes of personalized medicine and shared decision-making. Besides, it may be used easily in both clinical daily practice and research in patients with bipolar disorder.

## Supporting information

S1 File Data_k-means_anonymized. (XLS)
